# Evaluation of Antidiabetic Effects of the Traditional Medicinal Plant *Gynostemma pentaphyllum* and the Possible Mechanisms of Insulin Release

**DOI:** 10.1155/2015/120572

**Published:** 2015-06-23

**Authors:** Ezarul Faradianna Lokman, Harvest F. Gu, Wan Nazaimoon Wan Mohamud, Claes-Göran Östenson

**Affiliations:** ^1^Department of Molecular Medicine and Surgery, Karolinska Institutet, Karolinska University Hospital, SE-171 76 Stockholm, Sweden; ^2^Diabetes and Endocrine Unit, Cardiovascular, Diabetes and Nutrition Research Centre (CDNRC), Institute for Medical Research, Jalan Pahang, 50588 Kuala Lumpur, Malaysia

## Abstract

*Aims*. To evaluate the antidiabetic effects of* Gynostemma pentaphyllum *(*GP*) in Goto-Kakizaki (GK) rat, an animal model of type 2 diabetes, and to investigate the mechanisms of insulin release.* Methods*. Oral glucose tolerance test was performed and plasma insulin levels were measured.* Results*. An oral treatment with* GP *(0.3 g/kg of body weight daily) for two weeks in GK rats improved glucose tolerance versus placebo group (*P* < 0.01). Plasma insulin levels were significantly increased in the * GP*-treated group. The insulin release from* GP*-treated GK rats was 1.9-fold higher as compared to the control group (*P* < 0.001). * GP * stimulated insulin release in isolated GK rat islets at high glucose. Opening of ATP-sensitive potassium (K-ATP) channels by diazoxide and inhibition of calcium channels by nifedipine significantly decreased insulin response to* GP*. Furthermore, the protein kinase A (PKA) inhibitor H89 decreased the insulin response to* GP *(*P* < 0.05). In addition,* GP*-induced insulin secretion was decreased after preincubation of GK islets with pertussis toxin to inhibit exocytotic G_e_ proteins (*P* < 0.05).* Conclusion. *The antidiabetic effect of* GP *is associated with the stimulation of insulin release from the islets. * GP*-induced insulin release is partly mediated via K-ATP and L-type Ca^2+^ channels, the PKA system and also dependent on pertussis toxin sensitive G_e_-protein.

## 1. Introduction

The concern over efficacy and side effects of currently available therapies in the treatment of type 2 diabetes has promoted interest in discovery and development of antidiabetic drugs from traditional plants [1]. Several herbs with antidiabetic properties exert their effects by improving insulin secretion, glucose uptake by adipose and skeletal muscle tissues, or suppressing intestinal glucose absorption and hepatic glucose output [2].

*Gynostemma pentaphyllum *(*GP*) Makino, also known as Jiaogulan, is a climbing, perennial vine plant which grows in several parts of Asia countries including China, Vietnam, Japan, and Malaysia [3, 4]. Consumed as tea or food, the traditional uses of* GP* as a medicinal herb as claimed by practitioners have been pharmacologically and clinically proven in several studies.* GP* has been reported previously to have high radical scavenging capacity, antiproliferative, anti-inflammatory [3, 5, 6], hypoglycemic [7, 8], anticancer [9, 10], and antimicrobial effects [11].

The antidiabetic effects of* GP* have been clearly demonstrated in animal models of diabetes and randomly assigned type 2 diabetic patients. Thus, administration of* GP* tea in diabetic patients improves glucose tolerance by enhancing insulin sensitivity [12–14]. A similar effect was observed in the Goto-Kakizaki rat, an animal model of type 2 diabetes, showing that* GP* extract reduces the hepatic glucose output [15]. In other studies, an active gypenoside compound known as phanoside, isolated from* GP,* exhibited a potent insulin-releasing activity [16–18]. Furthermore, using diabetic rat models,* GP* saponins induce hypoglycemia, hypolipidemia, and immunocompetence effects associated with antioxidant activities [19, 20].

Several studies have been carried out to identify the antidiabetic effects of* GP*; however, these actions have not been elucidated in detail. In this study, the effects of* GP* water extract on GK rat blood glucose and serum insulin levels were studied. In addition,* GP* extract effects on mechanisms behind insulin secretion from GK rat islets were investigated.

## 2. Materials and Methods

### 2.1. Animals

Male spontaneous type 2 diabetic Goto-Kakizaki (GK) rats (150–350 g) were used in this study. GK rats, originating from Wistar (W) rats, were bred in our department. The rats were kept at 22°C with an alternating 12-hour light-dark cycle (6 am–6 pm) and were allowed access to food and water before being anesthetized for isolation of pancreatic islets. The study was approved by the Laboratory Animal Ethics Committee of the Karolinska Institutet.

### 2.2. Preparation of* Gynostemma pentaphyllum* (*GP*) Extract

*GP* water extracts were prepared according to standardized procedures from the whole herb obtained from China (Legosan AB, Kumla, Sweden). The standardized extract contained 98% gypenosides (German LEFO-Institut für Lebensmittel und Umwelt GmbH, Ahrensburg, Germany). The dried extract was suspended in ratio of 0.85 g to 20 mL in water for the use in the animal study and was prepared fresh daily.* GP* powder was diluted in distilled water and filtered with a 0.2 *μ*M filter.

### 2.3. Treatment with* GP*

The GK rats were divided into 2 groups: controls (*n* = 5) and* GP-*treated (*n* = 5). The rats were fasted overnight (14-15 hours), allowing access only to plain drinking water. For the treatment group,* GP* (0.3 g/kg of body weight) was administrated daily by oral gavage for 2 weeks whereas control group was given water only.

### 2.4. Oral Glucose Tolerance Test (OGTT)

An OGTT was performed to determine the effect of* GP* on blood glucose and insulin levels. Blood for glucose determination was obtained by tail-prick method at different time points: 0 minute (before glucose load of 0.2 g/100 g body weight), 30, 60, and 120 minutes. Blood glucose levels were measured using a glucometer, Accu-check Aviva (Roche Diagnostic GmbH, USA). Blood samples were also collected for the measurement of plasma insulin level at 0, 30, and 120 min. After two weeks' treatment with* GP*, the animals were killed using CO_2_. Pancreas was collected immediately for isolation of pancreatic islets and insulin secretion measurements.

### 2.5. Effect of Different Concentrations of* GP* on the Insulin Secretion from Isolated Islets

The isolation of islets was performed using collagenase method as described in previous protocols [16, 21, 22], and batch incubation of islets was performed as previously described [16, 21]. The medium used was Krebs-Ringer bicarbonate (KRB) buffer solution supplemented with 2 mg/mL of bovine albumin 10 mM HEPES and either 3.3 mM or 16.7 mM glucose. Following overnight incubation, the islets were preincubated at 3.3 mM glucose for 30–45 min at 37°C with an atmosphere of 5% CO_2_-95% air. Batches of three islets were incubated for 1 hour in 300 *μ*L Krebs-Ringer bicarbonate (KRB) buffer, at 37°C, with or without* GP* at different concentrations (1, 5, 10, and 15 mg/mL) at either 3.3 mM or 16.7 mM glucose. Aliquots obtained from batch incubations were further analyzed for insulin content using radioimmunoassay (RIA) [23].

### 2.6. Mechanisms of* GP*-Induced Insulin Release

To investigate how* GP* stimulates insulin release, we explored the ATP-sensitive potassium (KATP) channels. GK rat islets were incubated in Krebs-Ringer bicarbonate buffer (KRB) containing either 3.3 mM or 16.7 mM glucose. The medium was added with different incubation mixtures: 0.25 mM diazoxide (Sigma-Aldrich, USA) only (to open the K-ATP channels), 10 mg/mL of* GP* only, 50 mM of KCl (for depolarization of beta cells), and 10 mg/mL of* GP*. To investigate the effect of* GP* on L-type Ca^2+^ channels in beta cells, GK rat islets were incubated in KRB containing 16.7 mM glucose with addition of the L-type Ca^2+^ channel inhibitor, nifedipine (Sigma-Aldrich, USA). To investigate the effects of protein kinase A (PKA) and protein kinase C (PKC) on* GP*-induced insulin release, GK rat islets were incubated with or without* GP* and the PKA-inhibitor, H89 (10 *μ*M, Sigma-Aldrich, USA), or the PKC inhibitor, calphostin-C (1.5 *μ*M, Sigma-Aldrich, USA), for 60 min in KRB containing 3.3 mM and 16.7 mM glucose.

To assess the possible involvement of exocytotic G-protein, G_e_-protein, in* GP*-induced insulin release, GK rat islets were pretreated at 37°C overnight with 100 ng/mL pertussis toxin in RPMI 1640 culture medium (SVA, Sweden) containing 11 mM glucose, 30 mg* L*-glutamine, 10% heat-activated fetal calf serum, and antibiotics (100 IU/mL penicillin and 0.1 mg/mL streptomycin, Invitrogen, USA). After exposure overnight with or without pertussis toxin, islets were incubated with 10 mg/mL of* GP* at 3.3 mM and 16.7 mM glucose. Aliquots of the medium were analyzed for insulin content using RIA [23].

### 2.7. Statistical Analysis

The results are presented as mean ± SEM. Differences between experimental groups for OGTT and insulin secretion experiments were analyzed using unpaired *t*-test. The differences between means in the batch incubations and the mechanisms of insulin release via the K-ATP channels and Ca^2+^ channel as well as PKA and PKC mediators were analyzed for significance using one-way ANOVA, followed by Bonferroni's Multiple Comparison Post Hoc Test. All data were analyzed using Prism Graph Pad Software (CA, USA). A *P* value of less than 0.05 was considered to be significant.

## 3. Results

### 3.1. Oral Glucose Tolerance Test (OGTT)

**Figure 1:**
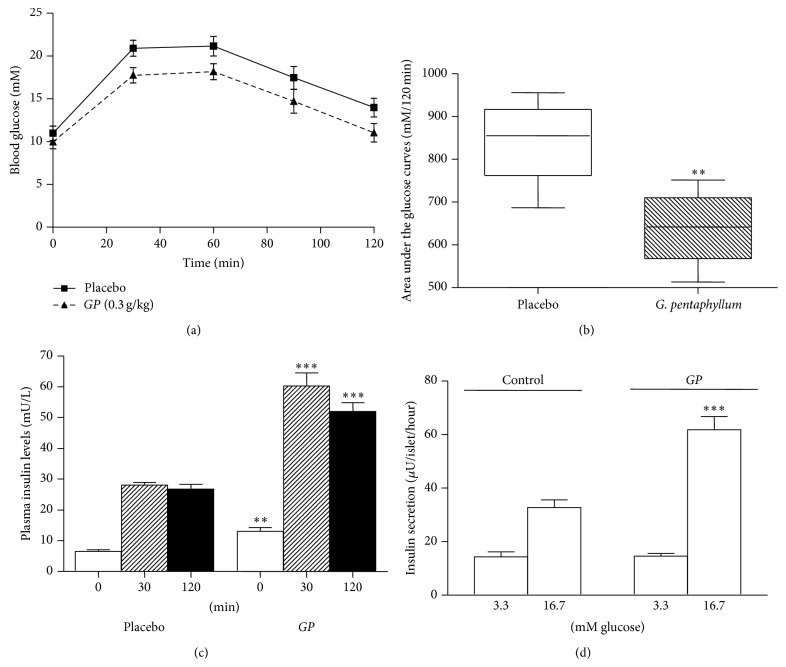
(a) The effects of treatment with* Gynostemma pentaphyllum* (*GP*) (0.3 g/kg of body weight) in GK rat (*n* = 5) on (a) blood glucose level in the oral glucose tolerance test,* GP*-treated (—) or placebo (—), (b) area under the glucose curves in the oral glucose tolerance test ^*∗∗*^ *P* < 0.01,* GP* versus Placebo. (c) Plasma insulin levels in* GP*-treated group compared to placebo group at 0, 30, and 120 min in the oral glucose tolerance test. ^*∗∗*^ *P* < 0.01 (*GP*-treated group versus placebo group at 0 min), ^*∗∗∗*^ *P* < 0.001 (*GP*-treated group versus placebo group at 30 and 120 min resp.). (d) Insulin secretion ^*∗∗∗*^ *P* < 0.001 (when compared with the placebo group at 16.7 mM glucose). All data are presented as means ± SEM and analyzed using unpaired *t*-test.

At baseline (day 0), glucose tolerance test was similar in both placebo and treatment groups with the area under the curves (AUCs) for glucose during 120 min (0–120 min) being 883.5 ± 63.4* versus *955.5 ± 74.1 mM, respectively. However, after two-week treatment with* GP*, glucose tolerance was significantly improved. The mean blood glucose levels at 120 min were found to decrease in the treated group as compared to the placebo group (11.0 ± 1.1* versus *13.9 ± 1.1 mM, resp.) (Figure 1(a)) with AUCs (0–120 min) being 639.6 ± 38.5* versus *842.4 ± 43.8 mM (*P* < 0.01) (Figure 1(b)). Plasma insulin levels after* GP* treatment were significantly increased both at 0 min (*P* < 0.01) and after glucose stimulation at 30 and 120 min (*P* < 0.001) when compared with the control group (Figure 1(c)). The body weights of* GP*- and placebo-treated GK rats showed comparable increase from day 0 to day 14 (data not shown).

### 3.2. Effects of* GP* on Insulin Secretion in GK Rat Islets

To identify if the oral administration of* GP* has an effect on the insulin release in the islets, the pancreas of the rats treated with* GP* for two weeks were collected (Figure 1(d)). At 3.3 mM glucose, insulin release was not significantly different between islets of GK rats treated with* GP* as compared to islets from control GK rats (14.6 ± 1* versus *14.3 ± 1.9 *μ*U/islet/hour). However, insulin release at 16.7 mM glucose was higher in islets from* GP-*treated rats as compared to islets from control rats (61.8 ± 4.9* versus *32.7 ± 2.8 *μ*U/islet/hour; *P* < 0.001).

**Figure 2:**
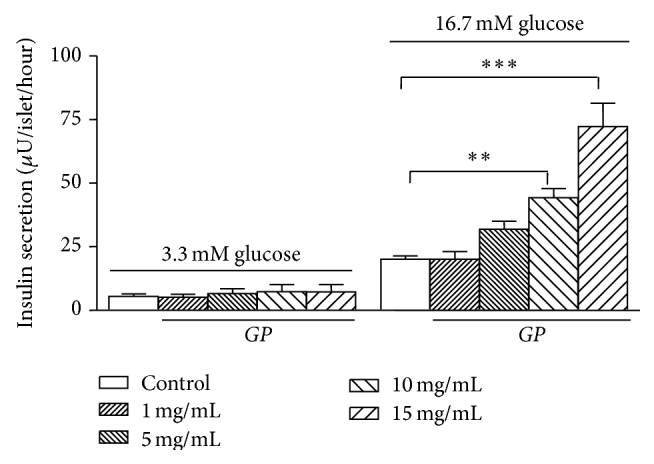
Effect of different concentrations of* Gynostemma pentaphyllum* (*GP*) (1,5, 10,15 mg/mL) on the insulin secretion in GK rat islets (*n* = 5). ^*∗∗*^ *P* < 0.01, ^*∗∗∗*^ *P* < 0.001 (when compared with the control group at 16.7 mM glucose only). Results of insulin release (*μ*U/islet/hour) are the means ± SEM of five independent experiments with three replicates for each experiment.

In separate* in vitro* experiments, isolated GK rat islets were incubated with different concentrations of* GP* to identify the insulin-stimulatory effect. At 3.3 mM glucose,* GP* at any concentration did not stimulate insulin release when compared to the control incubations (Figure 2). At 16.7 mM glucose, the addition of* GP* at 5, 10 and 15 mg/mL increased insulin release by 1.6, 2.2 (*P* < 0.01) and 3.6-fold (*P* < 0.001), respectively, when compared to the control group.

### 3.3. Effects of* GP* on the Exocytosis of Insulin Islets

**Figure 3:**
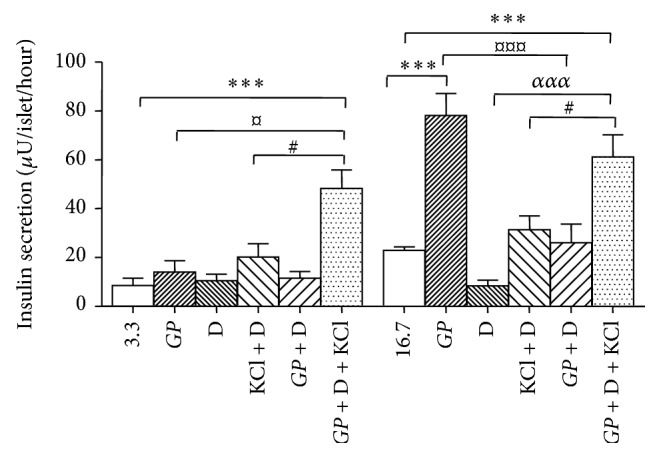
Effects of* Gynostemma pentaphyllum* (*GP*) on glucose stimulated insulin secretion from isolated GK rat islets with K-ATP channel opened by diazoxide (D) and depolarized by kalium chloride (KCl). ^*∗∗∗*^ *P* < 0.001 (when compared with control group with no addition at 3.3 mM and 16.7 mM glucose); ^#^ *P* < 0.05 (when compared with group with only D + KCl); ^¤^ *P* < 0.05, ^¤¤¤^ *P* < 0.001 (when compared with group with only* GP*); ^*ααα*^ *P* < 0.001 (when compared with D only). Results of insulin release (*μ*U/islet/hour) are the means ± SEM of five independent experiments with three replicates for each experiment.

The closure of K-ATP channels in pancreatic  *β*-cells leads to membrane depolarization and the stimulation of insulin release. Therefore, to understand if* GP* stimulates insulin release via the K-ATP channels, we used diazoxide. The addition of diazoxide inhibits insulin release by opening the K-ATP channels in pancreatic  *β*-cells [24]. KCl was used for membrane depolarization.* GP* significantly increased insulin release 3.4-fold (*P* < 0.001) compared to the control group at 16.7 mM glucose (Figure 3). The opening of K-ATP channels by adding diazoxide (0.25 mM) inhibited insulin release by 63% at 16.7 mM glucose. In addition, at 16.7 mM glucose, diazoxide decreased insulin response to* GP* from 78.1 ± 15.6 to 26 ± 17.0 *μ*U/islet/hour (*P* < 0.001). The addition of potassium chloride (KCl) to islets incubated with diazoxide to depolarize the  *β*-cells increased insulin release at both 3.3 mM and 16.7 mM glucose compared to the control group. At 16.7 mM, the insulin response to* GP* + diazoxide and KCl was significantly higher compared to islets incubated with diazoxide + KCl (at *P* < 0.05).

**Figure 4:**
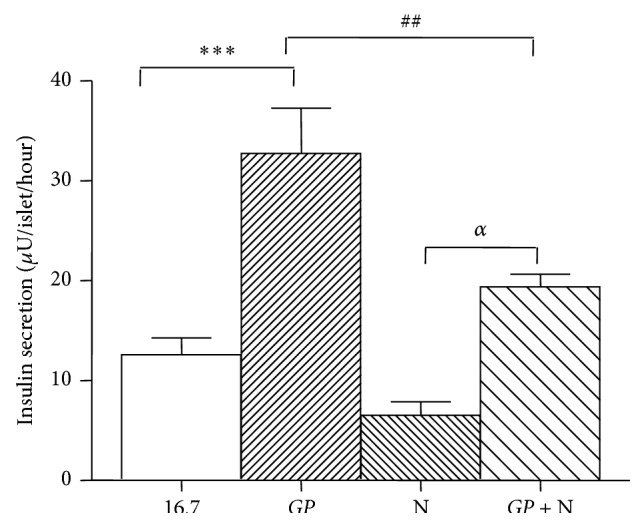
Effect of* Gynostemma pentaphyllum* (*GP*) with or without nifedipine (N) on glucose stimulated insulin secretion from isolated GK rat islets. ^*∗∗∗*^ *P* < 0.001 (when compared with control group with no addition); ^##^ *P* < 0.01 (when compared with group with only* GP*); ^*α*^ *P* < 0.05 (when compared with N). Results of insulin release (*μ*U/islet/hour) are the means ± SEM of six independent experiments with three replicates for each experiment.

To further understand if* GP* exerts its effect via L-type Ca^2+^ channel, nifedipine was used as an inhibitor of these channels [25]. At 16.7 mM glucose,* GP* stimulated insulin release by 2.6-fold (*P* < 0.001) compared to the control (Figure 4). The addition of nifedipine (10 *μ*M) decreased insulin release from 12.6 ± 1.7 to 6.5 ± 1.3 (*μ*U/islet/hour). Incubation with nifedipine and* GP* at 16.7 mM glucose decreased insulin secretion significantly compared to secretion induced by* GP* only, from 32.8 ± 4.5 to 19.4 ± 1.3 *μ*U/islet/hour (*P* < 0.01).

**Figure 5:**
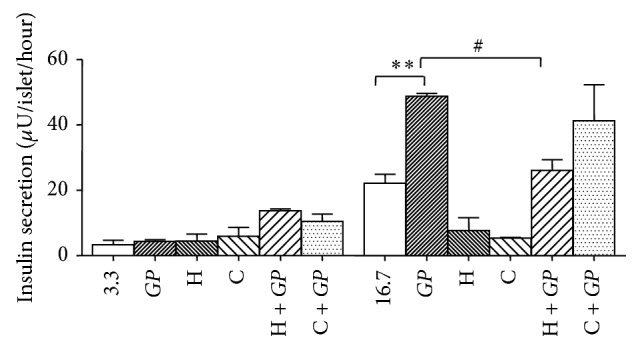
Effect of* Gynostemma pentaphyllum* (*GP*) with or without H89 and calphostin C (C) on glucose stimulated insulin secretion from isolated GK rat islets. ^*∗∗*^ *P* < 0.01 (when compared with control group with no addition); ^#^ *P* < 0.05 (when compared with group with only* GP*). Results of insulin release (*μ*U/islet/hour) are the means ± SEM of three independent experiments with three replicates for each experiment.

PKA or PKC pathways potentiate the insulin response to a metabolic stimulus [26]. To understand if* GP* exerts its effect via the PKA and PKC pathways, H89 (PKA inhibitor) and calphostin C (PKC inhibitor) were used. Incubation of islets with* GP *at 16.7 mM glucose stimulated insulin release by 2.2-fold (*P* < 0.01) (Figure 5). H89 (10 *μ*M) and calphostin C (10 *μ*M) decreased insulin release from 22.1 ± 2.8 to 7.7 ± 3.9 and to 5.4 ± 0.2 *μ*U/islet/hour, respectively, at 16.7 mM glucose compared to the control group. H89 decreased the insulin response to* GP* from 48.8 ± 0.9 to 26.07 ± 3.2 *μ*U/islet/hour (*P* < 0.05). Calphostin C inhibitor did not affect the insulin response to* GP* at 16.7 mM glucose.

**Figure 6:**
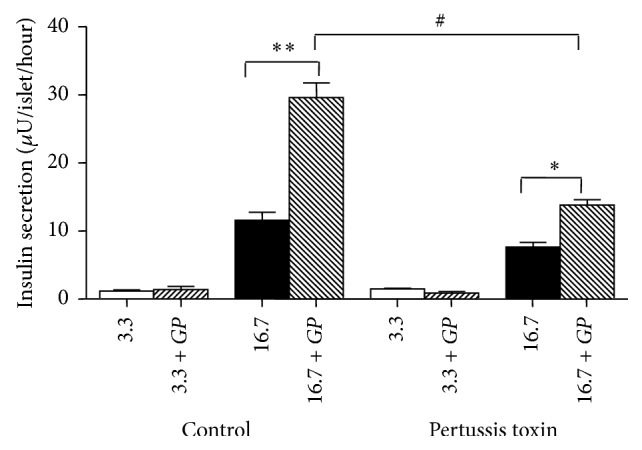
Effects of* Gynostemma pentaphyllum* (*GP*) on insulin secretion in GK rat islets with or without 24 hours incubation with 100 ng/mL of pertussis toxin. ^*∗*^ *P* < 0.05, ^*∗∗*^ *P* < 0.01 (when compared with control group with no addition at 16.7 mM glucose); ^#^ *P* < 0.05 (when compared with islets incubated with* GP* at 16.7 mM glucose without exposure to pertussis toxin) using paired *t*-test. Results of insulin release (*μ*U/islet/hour) are the means ± SEM of three independent experiments with three replicates for each experiment.

To understand if* GP* stimulates insulin release via exocytotic G_e_ proteins, pertussis toxin (PTX) was used as an inhibitor. PTX prevents the G proteins from interacting with their associated G protein-coupled receptors [27]. The preincubation of GK rat islets with pertussis toxin in the presence of* GP* at 11.1 mM glucose decreased the insulin secretion compared to the control group, from 29.6 ± 2.2 to 13.8 ± 0.7 *μ*U/islet/hour at 16.7 mM glucose (*P* < 0.05) (Figure 6).

## 4. Discussion

We have demonstrated treatment of GK rats, an animal model of type 2 diabetes, with* GP* extract for two weeks significantly improved glucose tolerance, increased plasma insulin levels, and increased insulin secretion from islets isolated from the treated rats. Furthermore, when tested* in vitro*, the* GP* extract stimulated insulin release from the isolated rat islets at high glucose only.* GP*-induced insulin release was partly mediated via K-ATP and L-type Ca^2+^ channels. The effects of* GP* were also mediated via the PKA system and partly dependent on pertussis toxin sensitive G_e_-protein at high glucose.

An oral administration of* GP* extract for 3 weeks in GK rats, which was done in the previous study, improved glucose tolerance and suppressed hepatic glucose output [15]. In addition to the effect of* GP* on the improvement of insulin sensitivity reported in the previous studies [12–15], the reduction of glucose levels observed in this study might be associated with a stimulatory effect on insulin release from the pancreatic islets. This observation is in concert with previous findings with phanoside, a compound isolated from* GP* [16–18]. The stimulated insulin secretion by* GP* is of importance since insulin controls the regulation of blood glucose level by increasing glucose uptake in the muscle and fat and suppressing glucose output in the liver [28].

Our findings have shown that* GP* has a potent insulin-stimulating activity in the GK rat islets. Therefore, to further understand the mechanisms behind* GP-*stimulated insulin release, we have explored the different sequences of pancreatic  *β*-cell stimulus-secretion coupling for glucose [16]. We first elucidated the role of the ATP-sensitive potassium (K-ATP) channels. In pancreatic  *β*-cells, the involvement of the K-ATP channel in regulation of glucose-dependent insulin secretion is a key factor. When blood glucose levels are elevated, glucose will be transported into the pancreatic  *β*-cell by GLUT2 glucose transporters and is then metabolized via glycolysis and glucose oxidation to ATP. The increase of ATP/ADP ratio in the  *β*-cells leads to closure of cell-surface ATP-sensitive K^+^ (K-ATP) channels. This in turn will lead to cell membrane depolarization, causing the opening of voltage-gated Ca^2+^ channels, cytosolic Ca^2+^ accumulation, and eventually insulin will be released [29–31]. In addition, activation of cAMP-dependent protein kinase PKA pathway is important for the insulin secretion signals in pancreatic  *β*-cells, whereas PKC signal pathways play an important role in potentiating the insulin response to glucose and incretins [26]. Our present findings suggest that* GP*-induced insulin secretion is modified by the PKA, but not the PKC, pathway.

Guanine nucleotide-binding proteins (G proteins) control several important sites regulating stimulus-secretion coupling and insulin secretion from pancreatic beta cells. G_s_-proteins mediate increases in intracellular cAMP associated with hormone-induced stimulation of insulin secretion, and G_i_-proteins mediate decreases in intracellular cAMP caused by inhibitors of insulin secretion, for example, epinephrine, somatostatin, prostaglandin E2, and galanin. G proteins also regulate ion channels, phospholipases, and distal sites in exocytosis, that is, by G_e_-proteins [32]. It seems plausible that* GP*, at least partly, exerts its stimulatory effects on insulin exocytosis, since preincubation of islets with the G-protein inhibitor pertussis toxin also decreased* GP*-induced insulin release. Our findings have shown that the effects of* GP*, at least to some extent, are most likely exerted by phanoside, a gypenoside that has been purified from* GP* [13–15]. Phanoside was also shown to exert its insulin-stimulatory effect distal to K-ATP channels and L-type Ca^2+^ channels, that is, mainly on the exocytotic machinery of the beta cells [16, 18].

## 5. Conclusions

We demonstrate that* GP* has antidiabetic properties mainly due to its stimulation of insulin release from the GK rat pancreatic islets.* GP*-induced insulin release is partly mediated via K-ATP and L-type Ca^2+^ channels. In addition,* GP* seems to interact with the PKA system and partially via exocytotic G_e_-proteins.
